# Impaired Glucocorticoid Receptor Dimerization Aggravates LPS-Induced Circulatory and Pulmonary Dysfunction

**DOI:** 10.3389/fimmu.2019.03152

**Published:** 2020-01-23

**Authors:** Martin Wepler, Jonathan M. Preuss, Tamara Merz, Clair Hartmann, Ulrich Wachter, Oscar McCook, Josef Vogt, Sandra Kress, Michael Gröger, Marina Fink, Angelika Scheuerle, Peter Möller, Enrico Calzia, Ute Burret, Peter Radermacher, Jan P. Tuckermann, Sabine Vettorazzi

**Affiliations:** ^1^Institute for Anesthesiologic Pathophysiology and Process Engineering, Ulm University, Ulm, Germany; ^2^Department of Anesthesiology, University Hospital, Ulm, Germany; ^3^Institute of Comparative Molecular Endocrinology (CME), Ulm University, Ulm, Germany; ^4^Institute of Pathology, University Hospital, Ulm, Germany

**Keywords:** glucocorticoid receptor, lung function, endotoxic shock, inflammation, osteopontin

## Abstract

**Background:** Sepsis, that can be modeled by LPS injections, as an acute systemic inflammation syndrome is the most common cause for acute lung injury (ALI). ALI induces acute respiratory failure leading to hypoxemia, which is often associated with multiple organ failure (MOF). During systemic inflammation, the hypothalamus-pituitary-adrenal axis (HPA) is activated and anti-inflammatory acting glucocorticoids (GCs) are released to overcome the inflammation. GCs activate the GC receptor (GR), which mediates its effects via a GR monomer or GR dimer. The detailed molecular mechanism of the GR in different inflammatory models and target genes that might be crucial for resolving inflammation is not completely identified. We previously observed that mice with attenuated GR dimerization (GR^dim/dim^) had a higher mortality in a non-resuscitated lipopolysaccharide (LPS)- and cecal ligation and puncture (CLP)-induced inflammation model and are refractory to exogenous GCs to ameliorate ALI during inflammation. Therefore, we hypothesized that impaired murine GR dimerization (GR^dim/dim^) would further impair organ function in LPS-induced systemic inflammation under human like intensive care management and investigated genes that are crucial for lung function in this setup.

**Methods:** Anesthetized GR^dim/dim^ and wildtype (GR^+/+^) mice were challenged with LPS (10 mg·kg^−1^, intraperitoneal) and underwent intensive care management (“lung-protective” mechanical ventilation, crystalloids, and norepinephrine) for 6 h. Lung mechanics and gas exchange were assessed together with systemic hemodynamics, acid-base status, and mitochondrial oxygen consumption (JO_2_). Western blots, immunohistochemistry, and real time quantitative polymerase chain reaction were performed to analyze lung tissue and inflammatory mediators were analyzed in plasma and lung tissue.

**Results:** When animals were challenged with LPS and subsequently resuscitated under intensive care treatment, GR^dim/dim^ mice had a higher mortality compared to GR^+/+^ mice, induced by an increased need of norepinephrine to achieve hemodynamic targets. After challenge with LPS, GR^dim/dim^ mice also displayed an aggravated ALI shown by a more pronounced impairment of gas exchange, lung mechanics and increased osteopontin (Opn) expression in lung tissue.

**Conclusion:** Impairment of GR dimerization aggravates systemic hypotension and impairs lung function during LPS-induced endotoxic shock in mice. We demonstrate that the GR dimer is an important mediator of hemodynamic stability and lung function, possibly through regulation of Opn, during LPS-induced systemic inflammation.

## Introduction

Anti-inflammatory acting glucocorticoids (GCs) mediate their effects through the glucocorticoid receptor (GR). The GR is an intracellular, ligand-activated transcription factor, which regulates gene transcription as a protein dimer or monomer in several mechanisms: as a protein dimer it can bind palindromic DNA sequences (glucocorticoid response elements—GRE) or DNA half sites as a monomeric protein [GR monomer, (1)]. Further GR monomers potentially inhibit the activity of pro-inflammatory transcription factors, e.g., NF-κB (2–5), AP-1 (6–8), or IRF-3 (9–11). This trans-repression is one major mechanism of GCs anti-inflammatory effects (12). However, it was recently shown that GC mediated anti-inflammatory responses also crucially require gene activation during inflammation (13). Indeed, mice with a point mutation in the GR DNA-binding domain (GR^dim^) exhibit less transactivation of GC-induced genes *in vivo* (1, 14) and fail to resolve inflammation in allergic (15), autoimmune (rheumatoid arthritis) (16), and systemic inflammation (17, 18).

Previously, we identified a novel mechanism by which GCs interfere with the pathogenesis of murine ALI, involving increased sphingosine kinase 1 (SphK1) gene expression and sphingosine-1-phosphate (S1P) production. The SphK1–S1P axis is recognized as an important regulator of endothelial barrier integrity that prevents lung inflammation (19, 20). In our previous study, we showed that the induction of SphK1 was GR dimerization dependent and therefore mice with an impaired GR dimerization (GR^dim/dim^) had an impaired lung barrier function during systemic lipopolysaccharides (LPS)-induced inflammation and GC treatment (13).

However, all studies described so far were lacking simultaneous control of temperature, as well as hemodynamic, and respiratory support, which is standard in intensive care treatment. Moreover, if potential beneficial effects of GCs in the treatment of lung injury occur, it is yet not clear which mechanisms are involved. Therefore, we tested the effects of an impairment of the GR in a murine model of LPS-induced systemic inflammation when factors like temperature, hemodynamics, and respiration are controlled (intensive care management including measurements of hemodynamics, infusion of crystalloids and norepinephrine to achieve hemodynamic targets, lung-protective mechanical ventilation, determination of gas exchange) in the present study. During intensive care management additional information about metabolic and hemodynamic parameters are observed that were missing in the former studies. We report that a congenital deficiency of the GR dimer aggravates hypotension, impairs lung function, and increases mortality in LPS-challenged mice.

## Materials and Methods

This study was approved by the federal authorities for animal research of the Regierungspräsidium Tübingen, Baden-Wuerttemberg, Germany, and performed in adherence with the National Institutes of Health Guidelines on the Use of Laboratory Animals and the European Union “Directive 2010/63 EU on the protection of animals used for scientific purposes”. GR^dim/dim^ mice (Nr3c1^tm3Gsc^) (21) were bred in a mixed background (129/SvEv × C57BL/6) and housed in the animal facility at University Ulm. GR^+/+^ littermate controls were used as wild-type mice. Animals were kept under standardized conditions and were equally distributed in terms of age and body weight.

### Implementation of General Anesthesia and Surgery

Surgery for all animals included induction of anesthesia with sevoflurane (2.5%; sevoflurane, Abbott, Wiesbaden, HE, Germany) as described previously (22, 23), followed by intraperitoneal injection (ip) of ketamine (120 μg·g^−1^; Ketanest-S, Pfizer, New York City, NY), midazolam (1.25 μg·g^−1^; Midazolam-ratiopharm, Ratiopharm, Ulm, BW, Germany), and fentanyl (0.25 μg·g^−1^; Fentanyl-hameln, Hameln Pharma Plus GmbH, Hameln, NI, Germany). Afterwards, animals were placed on a closed-loop-system for body temperature control (22, 23). Lung-protective mechanical ventilation using a small animal ventilator (FlexiVent, Scireq, MO, Canada) was performed via a tracheostomy (22, 23). Surgical instrumentation comprised catheters in the jugular vein, the carotid artery, and the bladder (22, 23). General anesthesia was titrated to guarantee complete tolerance against noxious stimuli and was sustained by continuous intravenous administration of ketamine, midazolam, and fentanyl to reach deep sedation. Animals were mechanically ventilated with ventilator settings being FiO_2_ 0.21%, respiratory rate 150·min^−1^, tidal volume of 6 mL·kg^−1^, and inspiratory/expiratory time ratio 1:2. Ventilation was modified to maintain an arterial PaCO_2_ between 30 and 45 mmHg, and positive end-expiratory pressure (PEEP) was adjusted according to the arterial PaO_2_ (PaO_2_/FiO_2_-ratio > 300 mmHg: PEEP = 3 cmH_2_O; PaO_2_/FiO_2_-ratio <300 mmHg: PEEP = 5 cmH_2_O; PaO_2_/FiO_2_-ratio <200 mmHg: PEEP = 8 cmH_2_O) (22, 23). Recruitment maneuvers (5 s hold at 18 cm H_2_O) were repeated hourly to avoid any impairment of thoraco-pulmonary compliance due to anesthesia- and/or supine position-induced atelectasis.

### Induction of Systemic Inflammation

After surgical instrumentation, systemic inflammation was induced by intraperitoneal (i.p.) injection of lipopolysaccharides (LPS = lipopolysaccharide from *Escherichia coli* [055:B5], L2880 Sigma, 10 mg·kg^−1^, dissolved in 10 μl·g^−1^ phosphate buffered saline [PBS]). Mice were then resuscitated with crystalloids (30 μl·g^−1^·h^−1^, Jonosteril, Braun Medical, Melsungen, HE, Germany). As soon as the mean arterial blood pressure (MAP) dropped below 55 mmHg, infusion of norepinephrine was started to reach a MAP >55 mmHg during the 6 h of resuscitation (maximum infusion rate 1.5 μg·h^−1^). If blood pressure declined despite increasing doses of norepinephrine, the experiment was terminated. GR^+/+^ mice, which received vehicle (10 μl·g^−1^ PBS) with subsequent resuscitation, served as controls.

### Parameters of Lung Mechanics, Hemodynamics, Gas Exchange, and Metabolism

Systemic hemodynamics, body temperature, and static thoraco-pulmonary compliance were recorded hourly. Blood gas tensions, acid-base status, glycaemia, and lactatemia were assessed at the end of the resuscitation period via aterial blood gas analysis (ABL800 Felx; Radiometer, Krefeld, Germany) (22, 23). At the end of the experiment, animals were exsanguinated, blood and lung tissue were taken immediately thereafter, and prepared for further analyses (22, 23). All lung tissue was utilized due to organ size. The left lung was harvested for histology and IHC, whereas the right lung served for immunoblotting, expression analysis, and cytokine and chemokine evaluation.

### Histological Analysis of Lung Tissue

Histological analysis of lung tissue was independently performed by two experienced pathologists (AS and PM) blinded for group assignment. Similar to previous studies (23), analyzed criteria comprised thickening of alveolar membranes, dystelectasis, emphysema, and inflammatory cell (lymphocytes) infiltration. These parameters were scored from 0 (absent), 1 (hardly detectable), 2 (rare), 3 (minor), 4 (moderate), to 5 (extensive).

### Mitochondrial Respiration

Mitochondrial respiratory capacity was determined via high-resolution respirometry with a clark-electrode-based system (Oxygraph 2k, OROBOROS Instruments Corp., Innsbruck, Austria) as described previously (22). Post-mortem heart, muscle, liver, and brain biopsies were mechanically homogenized in Mir05 (respiration medium). Mir05 is composed of 0.5 mM EGTA, 3 mM MgCl_2_·6H_2_O, 60 mM Lactobionic acid, 20 mM Taurine, 10 mM KH_2_PO_4_, 20 mM HEPES, 110 mM Sucrose, 1 g·L^−1^ bovine serum albumin). 1.5–2 mg of tissue (1.5 mg: heart, 2 mg tissue: muscle, liver, and brain) were added to the Oxygraph chamber. By addition of a defined sequence of substrates and inhibitors, various states of mitochondrial function could be assessed. Complex I activity was determined after addition of 10 mM pyruvate, 10 mM glutamate, 5 mM malate, and 5 mM ADP. Ten micrometers cytochrome c was added to check for mitochondrial integrity. Maximum oxidative phosphorylation (OxPhos) was evaluated after subsequent addition of 1 mM octanoyl-carnitine and 10 mM succinate. Leak compensation was assessed after inhibition of the ATP-synthase by 2.5 μM oligomycin, followed by stepwise titration of the uncoupling agent Carbonyl cyanide-4-(trifluoromethoxy)-phenylhydrazone (FCCP, final concentration 1.5 μM) to reach maximum respiratory activity of the electron transfer system in the uncoupled state (ETS).

### Western Blot

The lung was dissected at the end of the experiment and frozen on dry ice. The tissue was homogenized in EDTA-free lysis buffer with tissue homogenisator (Precellys^®^). Total protein concentration was determined using Pierce^®^ BCA Protein Assay Kit (23225). Proteins were separated by SDS-PAGE and blotted on nitrocellulose membrane using the Trans-Blot Turbo system (BioRad). Osteopontin was detected with anti-osteopontin primary antibody (mouse anti-mouse, LFMb-14, Santa Cruz Biotechnology Inc., sc-73631) diluted 1:500. Vincullin was detected as loading control using anti-vincullin primary antibody (mouse anti-mouse, Santa Cruz Biotechnology Inc., sc-73614) diluted 1:1.000. Primary antibody incubation was done overnight at 4°C. Secondary antibody (rabbit anti-mouse, HRP coupled polyclonal Ig, Dako, P0161) was diluted 1:10.000 and incubated 1 h at room temperature. Membrane blocking and dilutions were done with 5% BSA. Except for the last washing step which was done with 1x TBS, 0.1% TBST was used. Blots were developed using immobilion forte WBLUF0500 (Merck Millipore) and ImageLab software (version 5.2). Osteopontin abundancy was quantified using ImageJ (version 1.52a) by determination of mean signal intensity of osteopontin normalized to mean intensity of vincullin.

### Immunohistochemistry

The left lung was formalin-fixed and embedded in paraffin for immunohistochemistry analysis. Immunohistochemistry (IHC) for extravascular albumin content (anti-albumin rabbit polyclonal #16475-1-AP, Proteintech, USA) was performed as described previously (24). Primary antibodies were detected by secondary anti-rabbit antibody conjugated to AP (Alkaline Phosphatase-conjugated antibody; Jackson, ImmunoResearch, West Grove, Pa) and visualized with a red chromogen (Darko REAL Detection System Chromogen Red), and Mayers hematoxylin (Sigma, Taufkirchen, Germany). Visualization was performed using the Zeiss Axio Imager A1 microscope (Zeiss, Jena, TH, Germany). Four distinct 800.000 mm^2^ regions were quantified for intensity of signal by using the Axio Vision 4.8 software. Results are presented as mean densitometric sum red (24, 25).

### Cell Culture

The primary bone marrow-derived macrophages (BMDMs) were isolated from humerus, femur and tibia of 8–13 weeks old GR^+/+^ and GR^dim/dim^ mice as described previously (18). Briefly, cells were cultured until day 7 in DMEM (D5671, sigma) supplemented with 10% fetal bovine serum (FBS, F7524, sigma), 30% L929-cell conditioned medium, 1% Penicillin / Streptomycin (P0781, sigma), 1% L-Glutamine (G7513, sigma), 1% Sodium Pyruvate (S8636, sigma) at 37°C and 5% CO_2_. All BMDMs were treated with PBS as control and LPS (100 ng/ml, L6529, sigma) for the indicated time points. Osteopontin Elisa (R&D System) was performed with the supernatant of GR^+/+^ and GR^dim/dim^ BMDMs.

### Analysis of Relative mRNA Levels

For quantitative real-time PCR analysis (qRT-PCR), RNA was extracted from lungs by homogenization with tissue homogenisator (Precellys^®^) in Trizol (invitrogen) following the manufacturer's instructions. RNA quality was checked using the nanodrop (thermofisher). DNaseI-treated RNA (1 μg) was used to generate cDNA by oligo(dT) priming. qRT-PCR was performed with the ViiA™ 7 Realtime PCR System (Life technologies) using a Platinum SYBR Green (Invitrogen) and analyzed with the QuantStudio Realtime-PCR software using the ΔΔCT method. β*-Actin* and *Ribosomal protein L (Rpl*) served as housekeeping genes. The specific primers were obtained from Sigma with the sequences listed in Table 1.

**Table 1:** Specific primers for quantitative real-time PCR analysis (qRT-PCR).

**Gene**	**Forward primer**	**Reverse primer**
*β-Actin*	GCACCAGGGTGTGATGGTG	CCAGATCTTCTCCATGTCGTCC
*Il1-β*	GGCTGTGGAGAAGCTGTGGCA	GGGTCCGACAGCACGAGGCT
*Il-6*	AAACCGCTATGAAGTTCCTCTCTGC	AGCCTCCGACTTGTGAAGTGGT
*Il-10*	CAGAGCCACATGCTCCTAGA	TGTCCAGCTGGTCCTTTGTT
*Rpl*	CCTGCTGCTCTCAAGGTT	TGGCTGTCACTGCCTGGTACTT
*Tnfα*	AGGGGCCACCACGCTCTTCT	TGAGTGTGAGGGTCTGGGCCAT
*Sphkl*	CCAAGTGCACCCAAACTACC	GCCCCACCTTCTAGCTTTCT

### Measurements of Cytokine and Chemokine Concentrations

Bio-Plex Pro Mouse Cytokine 23-plex Assay (Group I) (Biorad) was used to measure 23 cytokines, chemokines and growth factors simultaneously in the plasma. The Bio-Plex Assay was conducted according to the manufacturer's protocol. The assay was performed with Bio-Plex 200 machine (Biorad) and analyzed with the Bio-Plex Manager TM 6.1 software (Biorad).

### Statistical Analysis

Unless stated otherwise, all data are presented as median (25th and 75th percentile). Data sets were analyzed using non-parametric statistics, i.e., Mann–Whitney *U*-test (one factor, two independent samples) or Kruskal–Wallis test with *post-hoc* Dunn's comparison testing (one factor, four independent samples). *P* < 0.05 were considered statistically significant. Quantitative graphical presentations and statistical analyses were accomplished by using GraphPad Prism 7 (GraphPad Software Inc., La Jolla, Calif).

## Results

### GR Dimerization Mediates Stability of Hemodynamics, Acid-Base Status, and Mitochondrial Respiration After LPS Challenge

**Figure 1:**
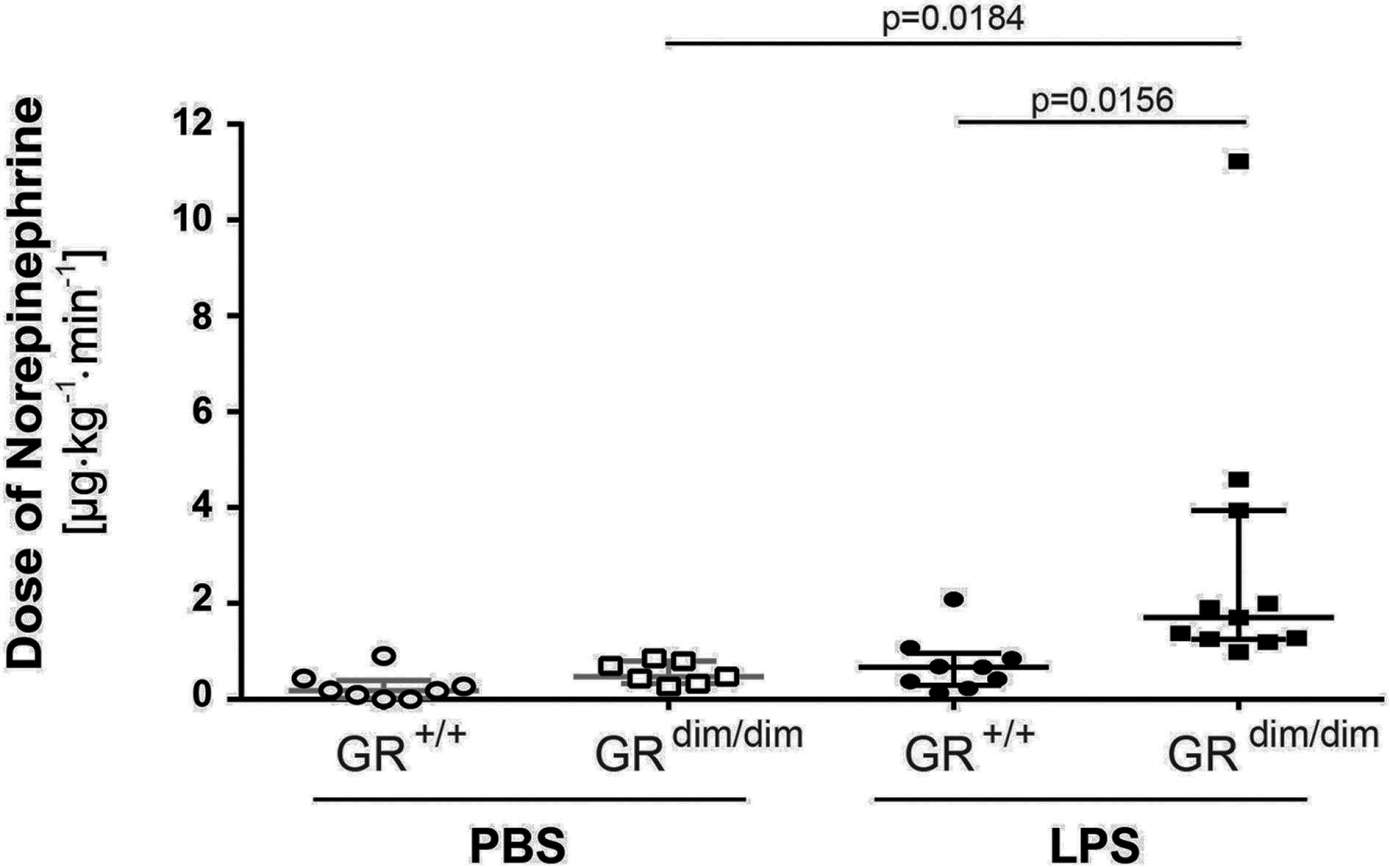
Doses of norepinephrine in mechanically ventilated GR^dim/dim^ and GR^+/+^ mice intraperitoneally challenged with lipopolysaccharides (LPS) or vehicle (PBS) and resuscitated (crystalloids, norepinephrine) for 6 h. Norepinephrine was titrated intravenously during resuscitation to keep systemic mean arterial blood pressure above 55 mmHg. LPS = lipopolysaccharide from *Escherichia coli* [055:B5], 10 mg·kg^−1^, dissolved in 10 μl·g^−1^ phosphate buffered saline (PBS). GR^+/+^ mice challenged with PBS: *n* = 8, GR^dim/dim^ mice challenged with PBS: *n* = 7, GR^+/+^ mice challenged with LPS: *n* = 9, GR^dim/dim^ mice challenged with LPS: *n* = 11. Data is presented as median (25th and 75th percentile and minimum/maximum).

**Figure 2:**
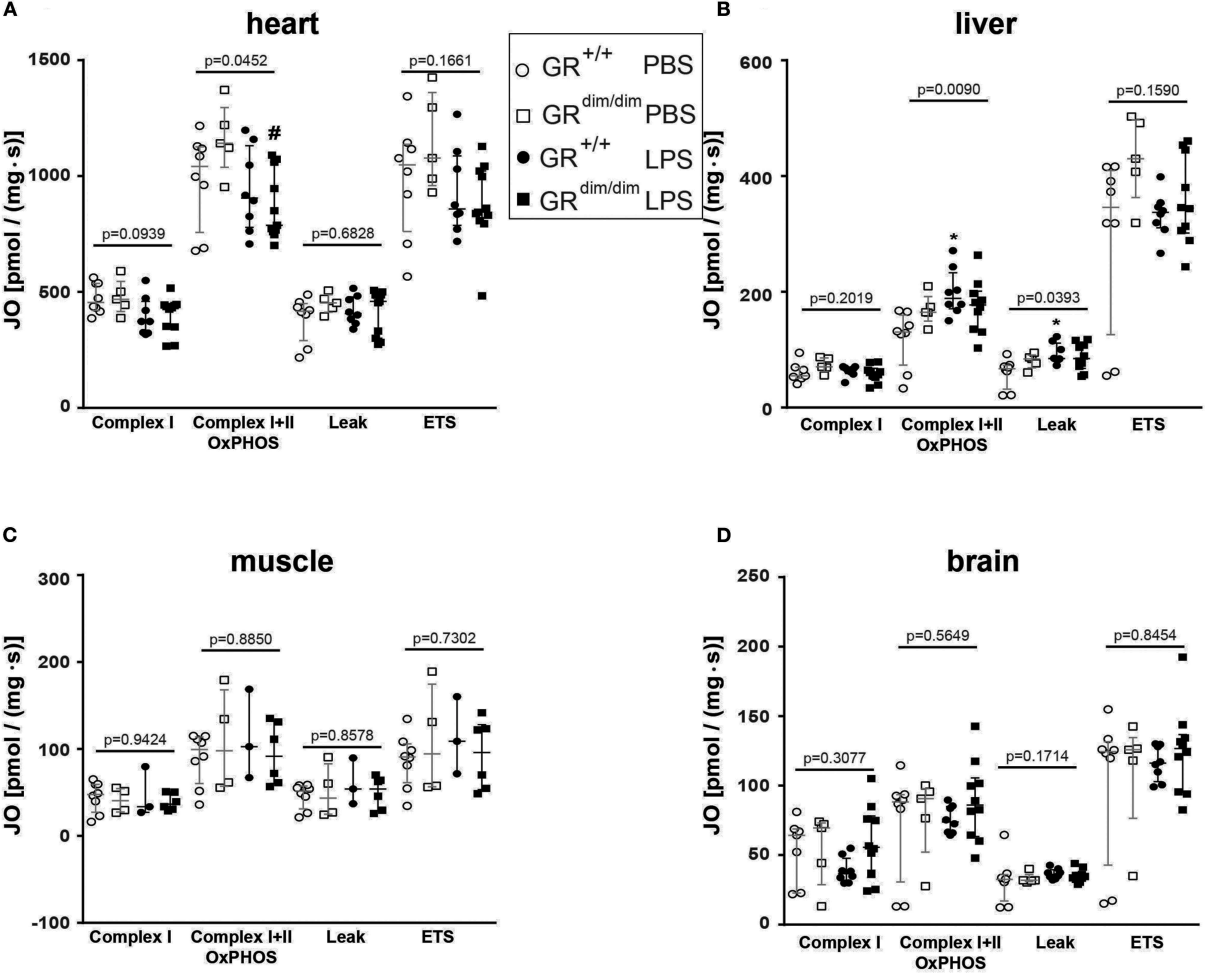
Mitochondrial respiration (JO_2_) in tissue from heart **(A)**, liver **(B)**, muscle **(C)**, and brain **(D)** in GR^dim/dim^ and GR^+/+^ mice intraperitoneal challenged with lipopolysaccharide (LPS) or vehicle (PBS). *p < 0.05 vs. GR^+/+^ PBS. ^#^*p* < 0.05 vs. GR^dim/dim^ PBS. Overall *p*-values from Kruskal–Wallis-Test are shown above accordingly. LPS = lipopolysaccharide from *Escherichia coli* [055:B5], 10 mg·kg^−1^, dissolved in 10 μl·g^−1^ phosphate buffered saline [PBS]. GR^+/+^ mice challenged with PBS: *n* = 7–8, GR^dim/dim^ mice challenged with PBS: *n* = 4–5, GR^+/+^ mice challenged with LPS: *n* = 3–8, GR^dim/dim^ mice challenged with LPS: *n* = 6–11. Data is presented as median (25th and 75th percentile and minimum/maximum).

To examine the effects of an impaired GR dimerization during resuscitation in LPS-induced systemic inflammation, hemodynamics, metabolic parameters, and mitochondrial respiration were investigated. Hemodynamic stability was defined as MAP >55 mmHg and preserved via infusion of crystalloids and, if necessary, norepinephrine (maximum infusion rate 1.5 μg·h^−1^). Therefore, in some mice (GR^+/+^ PBS *n* = 0, GR^dim/dim^ PBS *n* = 1, GR^+/+^ LPS *n* = 1, GR^dim/dim^ LPS *n* = 5), the experiment had to be terminated due to hemodynamic instability despite increasing norepinephrine doses, which lead to MAP values below 55 mmHg. After LPS-challenge, GR^dim/dim^ mice had a trend toward a lower MAP when compared to GR^+/+^ mice (Table 2). In line with the compromised systemic hemodynamics, norepinephrine requirements in GR^dim/dim^ mice challenged with LPS were significantly higher when compared to LPS-challenged GR^+/+^ animals (Figure 1 and Table 2). In GR^dim/dim^ mice, a challenge with LPS led to an increase of lactate levels at the end of the resuscitation phase, and, consequently, to a more pronounced decrease in base excess (BE) compared to GR^+/+^ mice, suggesting an aggravation of metabolic acidosis (Table 2). Because hyperlactatemia may be linked to disturbances in mitochondrial respiration (26, 27), important metabolic organs like muscle, heart, liver and brain were investigated for mitochondrial respiration in the current study. In LPS-challenged GR^dim/dim^ mice, oxygen flux during maximal coupled mitochondrial respiration (OxPhos) was decreased in heart tissue when compared to vehicle treated GR^dim/dim^ mice, whereas in GR^+/+^ animals OxPhos was not changed between PBS and LPS-challenge (Figure 2A), suggesting a disturbed mitochondrial function in the heart of GR^dim/dim^ animals. In the liver tissue, oxygen flux during maximal mitochondrial respiration and during inhibited ATP synthase in the coupled state (LEAK) was higher in LPS-challenged than in vehicle challenged GR^+/+^ mice (Figure 2B), suggesting an increased mitochondrial respiratory capacity in these animals. However, in the muscle and brain mitochondrial respiration was not changed neither in GR^dim/dim^ nor in GR^+/+^ animals during PBS- or LPS-challenge under resuscitation (Figures 2C,D). In summary, the impaired dimerization of the GR results in higher noradrenaline requirements and therefore compromised systemic hemodynamics as well as a more pronounced lactic acidosis and altered mitochondrial respiration during LPS-challenge.

**Table 2:** Hemodynamic and metabolic measurements as well as parameters of lung function in GR^dim/dim^ and GR^+/+^ mice intraperitoneally challenged with lipopolysaccharide (LPS) or vehicle (phosphate buffered saline, PBS) at the end of the experiment.

**Parameters**	**Resuscitation (IV crystalloids and norepinephrine)**			
	**GR^**+/+**^** **+** **Vehicle** **(*n* = 8)**	**GR^**dim/dim**^** **+** **Vehicle** **(*n* = 7)**	**GR^**+/+**^** **+** **LPS** **(*n* = 9)**	**GR^**dim/dim**^** **+** **LPS** **(*n* = 11)**
Bodyweight [g]	26.7 (23.0; 31.6)	24.6 (20.6; 32.7)	29.0 (26.6; 31.1)	24.0 (22.7; 28.2)
Heart rate [beats·min^−1^]	478 (335; 518)	551 (520; 558)	529 (471; 557)	523 (460; 580)
Mean arterial pressure [mmHg]	61 (56; 65)	54 (52; 61)	56 (49; 58)	49 (43; 51)
PaCO_2_ [mmHg]	37 (30; 45)	41 (39; 44)	34 (33; 47)	43 (40; 46)
Minute ventilation [mL·kg^−1^·min^−1^]	905 (838; 1,058)	1,000 (980; 1,080)	1,000 (960; 1,040)	1,060 (980; 1,170)
Horovitz-Index [mmHg]	465 (409; 498)	363 (342; 371)	349 (302; 463)	327 (291; 352)
Glucose [mg·dL^−1^]	129 (121; 147)	139 (136; 176)	78 (60; 89)	94 (80; 183)
Arterial pH	7.28 (7.26; 7.34)	7.23 (7.19; 7.28)	7.14 (7.12; 7.32)	7.12 (6.99;7.20)
Arterial base excess [mmol·L^−1^]	−8.4 (−11.3; −5.6)	−7.9 (−10.5; −7.4)	−11.0 (−13.2; −10.2)	–**14.5 (**–**19.9;** –**12.0)**^**#**^
Lactate [mmol·L^−1^]	1.1 (0.8; 1.6)	1.5 (1.2; 2.1)	2.8 (2.1; 3.1)	**5.3 (3.9; 6.6)#**
Hemoglobin [g·dL^−1^]	8.7 (8.3; 10.6)	9.0 (8.6; 11.4)	8.2 (7.3; 10.1)	9.0 (8.0; 9.7)
Urinary output [μL]	1,796 (997; 2,413)	2,309 (1,494; 3,628)	881 (646; 1,906)	**969 (514; 1,060)^#^**

# *P <0.05 vs. GR^dim^ PBS. PBS = phosphate buffered saline (vehicle), 10 μl·g^−1^. LPS = Lipopolysaccharide from Escherichia coli (055:B5), 10 mg·kg^−1^. Data is shown as median (25th and 75th percentile)*.

### GR^dim/dim^ Mice Have Higher Mortality After LPS Challenge With Subsequent Resuscitation

In LPS-challenged mice with subsequent resuscitation for a maximum of 6 h (lung-protective mechanical ventilation, hemodynamic measurements, crystalloid and norepinephrine infusion to keep hemodynamic stability), mortality was significantly higher in GR^dim/dim^ mice when compared to GR^+/+^ animals (Supplementary Figure 1).

### No Differences in Most Plasma Cytokines 6 h After LPS Challenge and Resuscitation

After LPS-challenge, the concentration of Il-1α in plasma increased in GR^dim/dim^, but not in GR^+/+^ mice. In contrast, concentrations of Il-2, Eotaxin, and Ifnγ only increased in GR^+/+^ mice, but not in GR^dim/dim^ mice, however, all are expressed on a low level (Table 3). Pro-inflammatory plasma cytokines like Il1-β (Figure 3A), Il-6 (Figure 3B), and Tnfα (Figure 3C) were significantly induced in both genotypes after LPS challenge, demonstrating that the intensive care management and resuscitation does not increase basal level of these inflammatory cytokines.

**Table 3:** Concentrations of cytokines in plasma of GR^dim/dim^ and GR^+/+^ mice intrapoeritoneal challenged with lipopolysaccharide (LPS) or vehicle (phosphate buffered saline, PBS) measured at the end of the experiment.

**Parameters**	**Resuscitation (IV crystalloids and norepinephrine)**			
	**GR^**+/+**^ + Vehicle (*n* = 7–8)**	**GR^**dim/dim**^ + Vehicle (*n* = 6–7)**	**GR^**+/+**^ + LPS (*n* = 7–8)**	**GR^**dim/dim**^ + LPS (*n* = 10–11)**
Il-1 alpha [pg·ml^−1^]	27 (12; 65)	19 (10; 22)	67 (55; 70)	**67 (22; 68)^#^**
Il-2 [pg·ml^−1^]	3.5 (1.1; 6.4)	2.9 (1.6; 6.6)	16.2 (14.0; 19.2)^*^	13.7 (7,5; 17.3)
Il-3 [pg·ml^−1^]	1.4 (0.8; 2.0)	1.1 (0.2; 1.6)	13.1 (12.4; 14.0)^*^	**12.7 (5.4; 13.7)^#^**
Il-5 [pg·ml^−1^]	15.9 (7.6; 27.9)	12.8 (6.7; 28.0)	23.5 (19.2; 31.1)	29.5 (18.1; 48.0)
Il-10 [pg·ml^−1^]	27 (20; 48)	22 (16; 52)	336 (301; 487)^*^	**1,017 (428; 1,416)^#^**
Eotaxin [pg·ml^−1^]	602 (517; 736)	857 (775; 1,292)	1,756 (1,263; 1,992)^*^	1,365 (1,000; 1,779)
KC [pg·ml^−1^]	30 (24; 38)	26 (19; 32)	2,318 (1,454; 2,698)^*^	**1,358 (266; 2,268)^#^**
Mcp-1 [pg·ml^−1^]	289 (239; 349)	239 (194; 398)	51,720 (30,400; 96,096)^*^	**78,358 (4,385; 164,779)^#^**
Rantes [mg·dL^−1^]	72 (70; 80)	51 (33; 59)	4,252 (2,936; 6,277)^*^	**3,457 (583; 4,424)^#^**
Ifn gamma [pg·ml^−1^]	6 (3; 10)	13 (6; 26)	90 (45; 195)^*^	107 (16; 152)

* *P <0.05 vs. GR^+/+^ PBS*.

# *P <0.05 vs. GR^dim^ PBS. PBS = phosphate buffered saline (vehicle), 10 μl·g^−1^. LPS = Lipopolysaccharide from Escherichia coli (055:B5), 10 mg·kg^−1^. Data is shown as median (25th and 75th percentile). Bold values indicate significant differences*.

**Figure 3:**
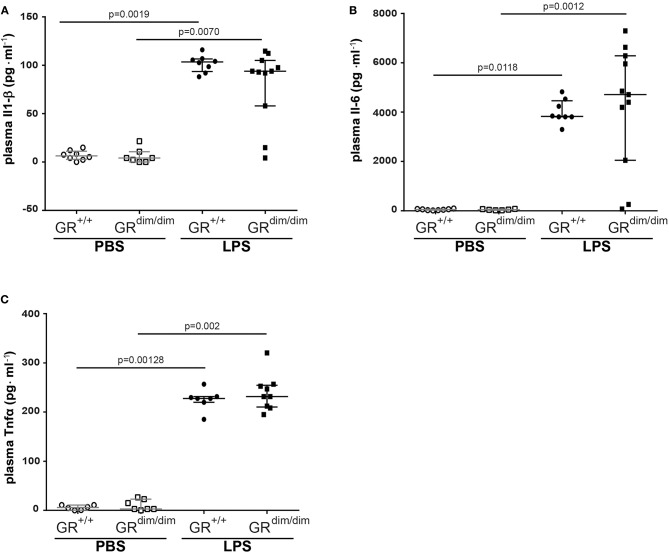
Level of inflammatory cytokines in the plasma. Concentration of **(A)** interleukin 6 (Il-6), **(B)** interleukin 1 β (Il-1β), and **(C)** tumor necrosis factor α (Tnfα) in the plasma of GR^dim/dim^ and GR^+/+^ mice after an intraperitoneally challenge with lipopolysaccharides (LPS) or treatment with PBS. LPS = lipopolysaccharide from *Escherichia coli* [055:B5], 10 mg·kg^−1^, dissolved in 10 μl·g^−1^ phosphate buffered saline (PBS). GR^+/+^ mice challenged with PBS: *n* = 6–8, GR^dim/dim^ mice challenged with PBS: *n* = 6–7, GR^+/+^ mice challenged with LPS: *n* = 7–8, GR^dim/dim^ mice challenged with LPS: *n* = 9–11. Data is presented as median (25th and 75th percentile and minimum/maximum).

### Lung Function Is Impaired in GR^dim/dim^ Mice During Intensive Care Treatment

**Figure 4:**
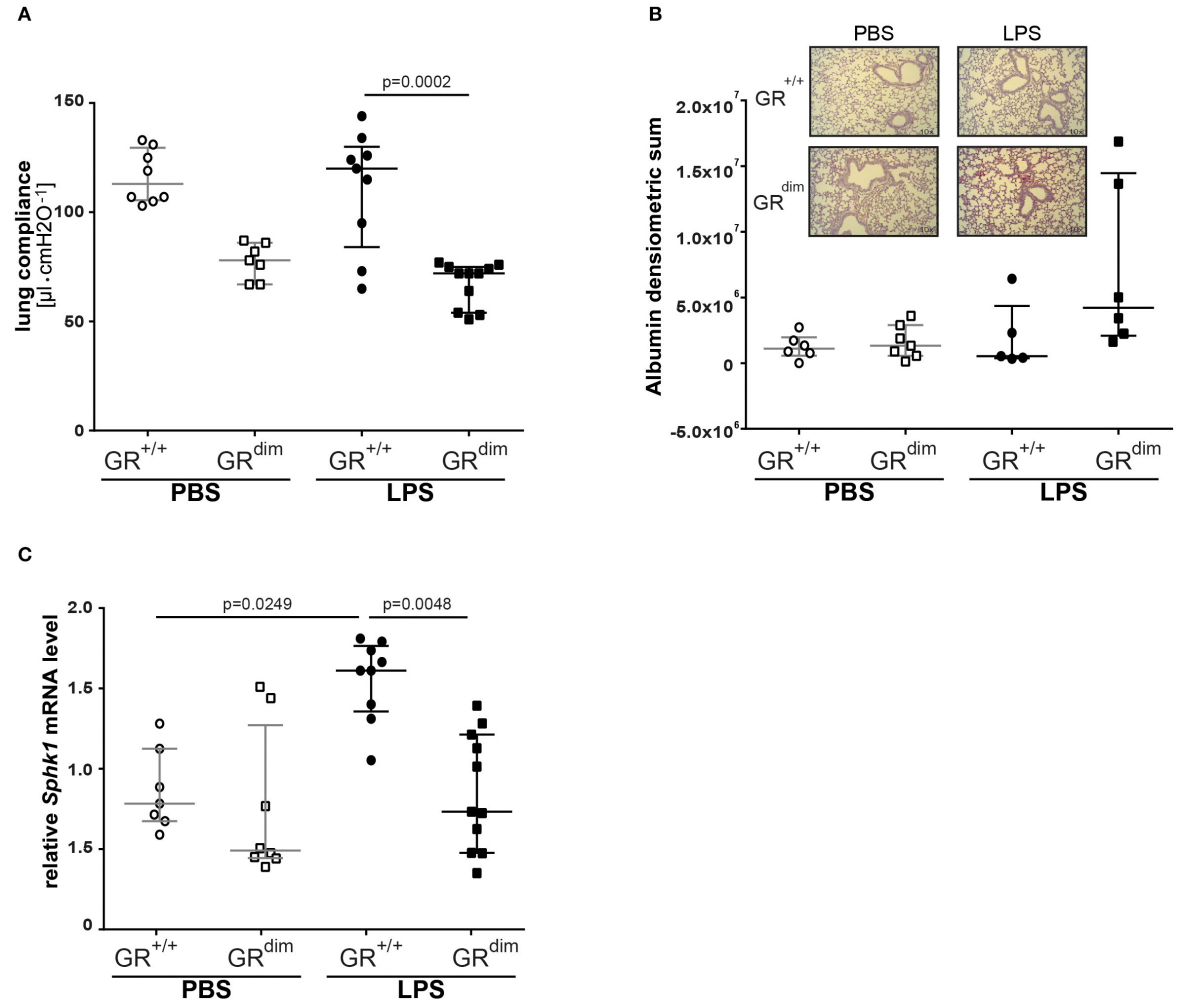
Lung function and mRNA expression of glucocorticoid receptor associated genes in mechanically ventilated GR^dim/dim^ and GR^+/+^ mice intraperitoneally challenged with lipopolysaccharides (LPS) or vehicle (PBS). **(A)** Lung compliance: *p* = 0.0735 for GR^+/+^ vs. GR^dim/dim^ mice treated with PBS for Kruskal–Wallis-Test with multiple comparisons and *post-hoc* Dunn's Test. LPS = lipopolysaccharide from *Escherichia coli* [055:B5], 10 mg·kg^−1^, dissolved in 10 μl·g^−1^ phosphate buffered saline (PBS). GR^+/+^ mice challenged with PBS: *n* = 8, GR^dim/dim^ mice challenged with PBS: *n* = 7, GR^+/+^ mice challenged with LPS: *n* = 9, GR^dim/dim^ mice challenged with LPS: *n* = 11. Data is presented as median (25th and 75th percentile and minimum/maximum). **(B)** Immunohistochemical (IHC) analysis of extravascular albumin expression in lung tissue of GR^dim/dim^ and GR^+/+^ mice challenged with lipopolysaccharide (LPS) or vehicle (PBS) and example pictures of albumin extravasation in lung tissue in GR^+/+^ and GR^dim/dim^ mice (lower picture). Albumin was detected with a secondary Alkaline Phosphatase-conjugated antibody and visualized with a red chromogen and regions were quantified for signal. GR^+/+^ mice challenged with PBS: *n* = 6, GR^dim/dim^ mice challenged with PBS: *n* = 7, GR^+/+^ mice challenged with LPS: *n* = 5, GR^dim/dim^ mice challenged with LPS: *n* = 6. Data is presented as median (25th and 75th percentile and minimum/maximum). **(C)** Measurements of relative mRNA level of sphingosine kinase 1 (*Sphk1*) in lung tissue of GR^dim/dim^ and GR^+/+^ mice challenged with lipopolysaccharide (LPS) or vehicle (PBS). GR^+/+^ mice challenged with PBS: *n* = 7, GR^dim/dim^ mice challenged with PBS: *n* = 8, GR^+/+^ mice challenged with LPS: *n* = 9, GR^dim/dim^ mice challenged with LPS: *n* = 11. Data is presented as median (25th and 75th percentile and minimum/maximum).

Lung compliance as a marker for lung mechanics was reduced in PBS-treated GR^dim/dim^ mice in comparison to PBS-treated GR^+/+^ animals (Figure 4A). A significantly lower lung compliance was observed in GR^dim/dim^ mice compared to GR^+/+^ controls during LPS-challenge (Figure 4A). In line with a lower lung compliance, GR^dim/dim^ mice had a reduced Horovitz-Index (partial pressure of oxygen in the arterial blood, divided by the inspiratory concentration of oxygen) as a marker for systemic oxygenation (Table 2). However, GR^+/+^ animals did not show any significant changes in lung compliance or Horovitz-Index after LPS-challenge in comparison to PBS-challenged GR^+/+^ animals (Figure 4A and Table 2), suggesting a more severe lung dysfunction in response to LPS-challenge in GR dimerization impaired mice. The histological evaluation of lung tissue revealed no significant differences in the total score between the corresponding groups, however the GR^dim/dim^ animals had a slight elevated total score (Table 4). Immunohistochemistry (IHC) for albumin extravasation showed a higher expression in lung tissue after LPS-challenge in GR^dim/dim^ mice compared to GR^+/+^ mice, whereas no difference was observed in extravascular albumin expression between GR^dim/dim^ and GR^+/+^ mice after vehicle treatment (Figure 4B). In previous studies, increased albumin extravasation and vascular leakage was accompanied by a reduced expression of *Sphk1* dependent on the GR dimerization during inflammation (13). In the present study, LPS-challenged GR^+/+^ mice showed increased *Sphk1* expression that correlated with lower albumin expression compared to GR^dim/dim^ challenged LPS mice having increased extravascular albumin expression and significantly reduced *Sphk1* expression (Figures 4B,C). Taken together, lung function was impaired in GR^dim/dim^ mice upon LPS-challenge during intensive care treatment. In PBS-treated animals a so far not described slight basal difference in lung function was observed in GR^dim/dim^ mice. These data revealed that the dimerization of the GR is crucial for the lung compliance in an inflammatory setting under intensive care management.

**Table 4:** Quantification of lung histology analysis in GR^dim/dim^ and GR^+/+^ mice that underwent an intraperitoneally challenge with lipopolysaccharide (LPS) or vehicle (phosphate buffered saline, PBS) and were resuscitated for 6 h thereafter.

**Parameters**	**Resuscitation (IV crystalloids and norepinephrine)**			
	**GR^**+/+**^** **+** **Vehicle** **(*n* = 8)**	**GR^**dim/dim**^** **+** **Vehicle** **(*n* = 7)**	**GR^**+/+**^** **+** **LPS** **(*n* = 5)**	**GR^**dim/dim**^** **+** **LPS** **(*n* = 6)**
Alveolar membrane thickening	1.0 (0.9; 1.0)	1.5 (1.0; 2.0)	1.0 (1.0; 2.0)	1.5 (1.0; 2.0)
Dystelectasis	0.5 (0.0; 0.5)	0.5 (0.0; 1.0)	0.0 (0.0; 0.0)	0.3 (0.0; 0.5)
Emphysema	2.0 (2.0; 2.0)	2.0 (1.8; 2.0)	2.5 (2.5; 2.5)	2.5 (2.0; 3.0)
Lymphocytes	1.0 (1.0; 1.3)	2.0 (2.0; 2.5)^*^	1.0 (1.0; 2.0)	2.0 (1.3; 2.0)
Total score	4.5 (4.0; 5.0)	6.0 (5.8; 7.0)	4.5 (4.5; 6.5)	5.8 (5.1; 6.8)

*For detailed description of the score for histological tissue analysis see Methods section*.

* *P <0.05 vs. GR^+/+^ vehicle. PBS = phosphate buffered saline (vehicle), LPS = Lipopolysaccharide from Escherichia coli (055:B5), 10 mg·kg^−1^. Data is shown as median (interquartile range)*.

### Increased Osteopontin in GR^dim/dim^ Mice Might Contribute to Disturbed Lung Function

**Figure 5:**
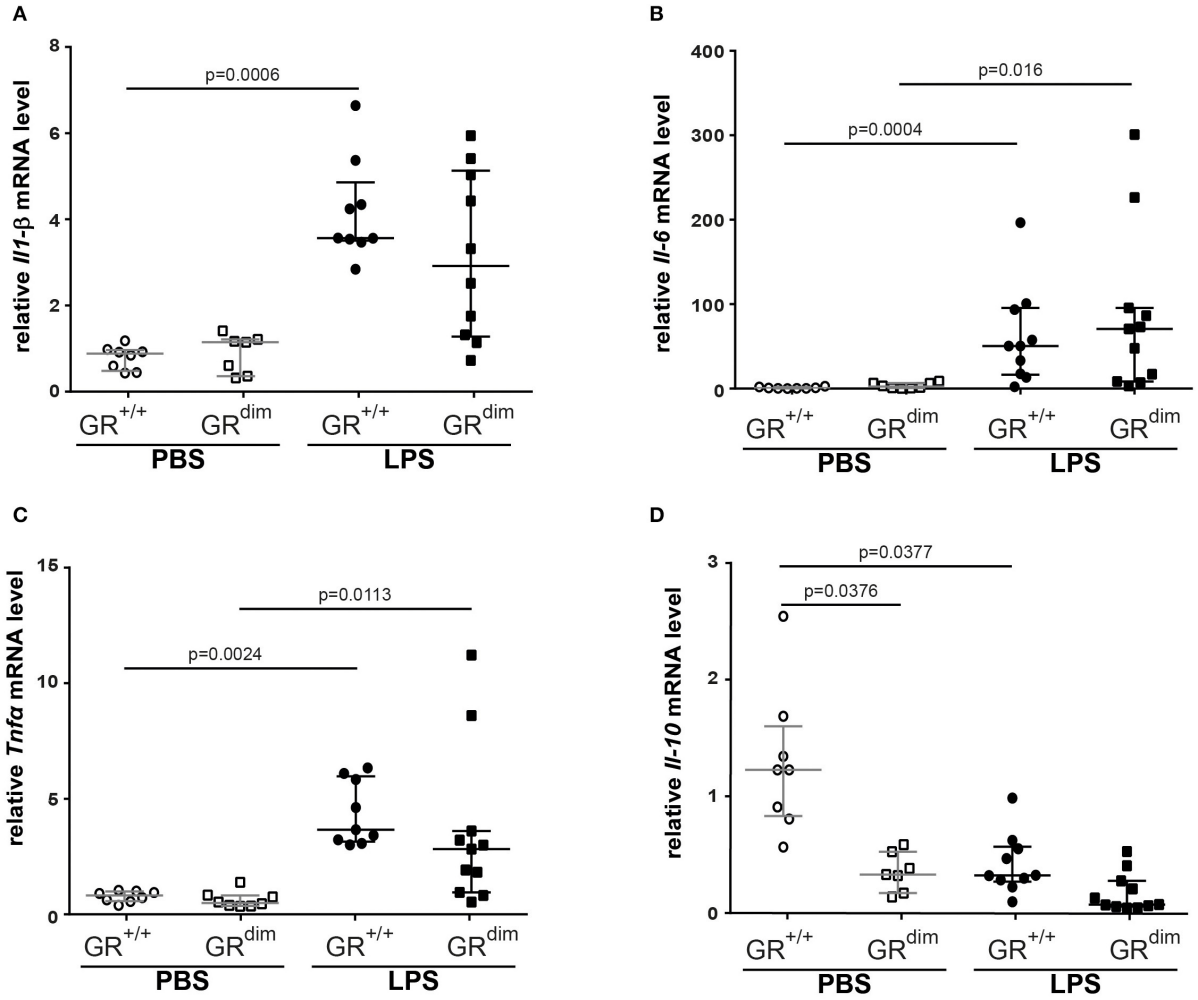
Measurements of relative mRNA level of cytokines in lung tissue. Relative mRNA level of **(A)** interleukin 1β (*Il-1*β), **(B)** interleukin 6 (*Il-6*), **(C)** tumor necrosis factor α (*Tnf*α), and **(D)** interleukin 10 (*Il-10)* in lung tissue of GR^dim/dim^ and GR^+/+^ mice intraperitoneally challenged with lipopolysaccharide (LPS) or vehicle (PBS). LPS = lipopolysaccharide from *Escherichia coli* [055:B5], 10 mg·kg^−1^, dissolved in 10 μl·g^−1^ phosphate buffered saline (PBS). GR^+/+^ mice challenged with PBS: *n* = 8, GR^dim/dim^ mice challenged with PBS: *n* = 7–8, GR^+/+^ mice challenged with LPS: *n* = 9–10, GR^dim/dim^ mice challenged with LPS: *n* = 10–11. Data is presented as median (25th and 75th percentile and minimum/maximum).

**Figure 6:**
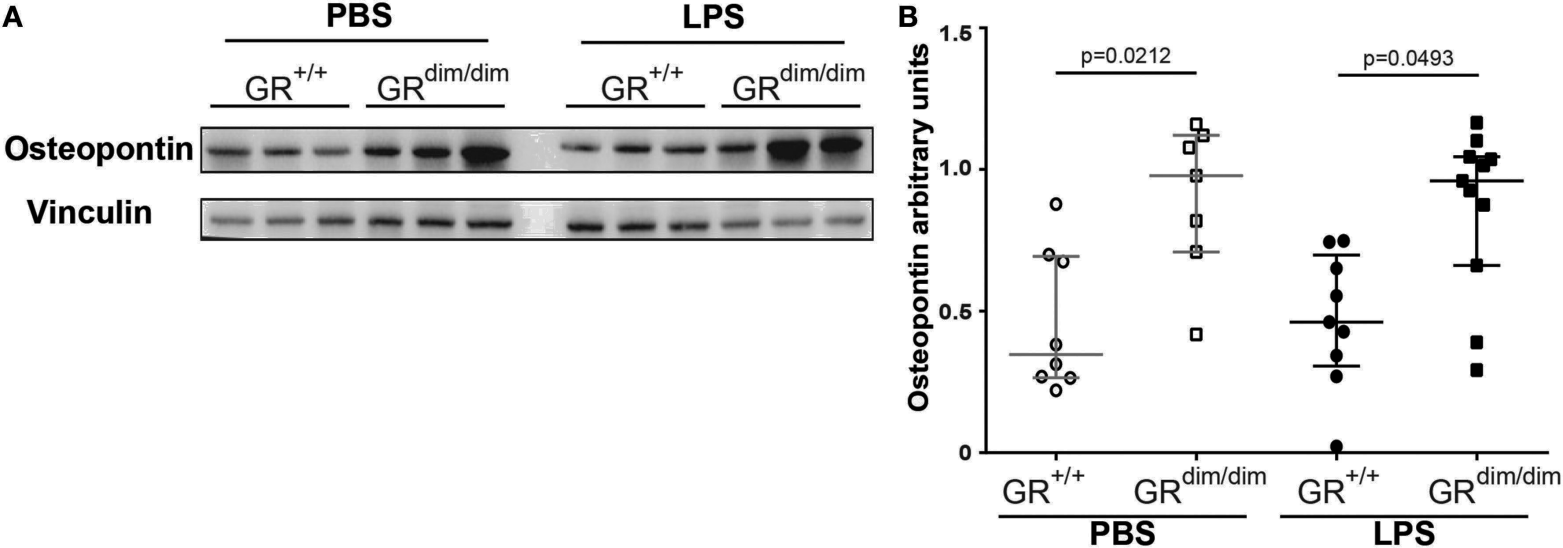
Analysis of Osteopontin (Opn) levels in lung tissue. **(A)** Representative western blot analysis and **(B)** arbitrary units of Opn, in lung tissue of GR^dim/dim^ and GR^+/+^ mice intraperitoneally challenged with lipopolysaccharide (LPS) or vehicle (PBS). LPS = lipopolysaccharide from *Escherichia coli* [055:B5], 10 mg·kg^−1^, dissolved in 10 μl·g^−1^ phosphate buffered saline (PBS). GR^+/+^ mice challenged with PBS: *n* = 8, GR^dim/dim^ mice challenged with PBS: *n* = 7, GR^+/+^ mice challenged with LPS: *n* = 9, GR^dim/dim^ mice challenged with LPS: *n* = 11. Data is presented as median (25th and 75th percentile and minimum/maximum).

Inflammatory cytokine mRNA expression of *Il1-*β (Figure 5A), *Il-6* (Figure 5B), *Tnf*α (Figure 5C), and *Il-10* (Figure 5D) in the lung revealed no significant differences between GR^dim/dim^ and GR^+/+^ mice after challenge with LPS under intensive care management. Therefore, the reduced lung compliance is most likely not a result of the aforementioned inflammatory mediators. Next to the expression of *Sphk1*, which was identified as an important regulator of lung barrier integrity, we now also aimed at other potential regulators of lung injury. Osteopontin (Opn, secreted phosphoprotein 1–Spp1) is a crucial mediator for inflammatory responses and a regulator of inflammation, especially lung inflammation. Opn neutralizing antibody could protect mice against ALI during sepsis (28). In our setting (intensive care management) Opn protein expression was significantly enhanced in lungs of GR^dim/dim^ compared to GR^+/+^ mice, both under PBS-treatment and LPS-challenge (Figures 6A,B). Opn was shown to have an impact on type-1 immunity to bacterial infections as OPN deficient mice have increased Il-10 production (29). In accordance with this, GR^dim/dim^ mice with increased levels of Opn showed a trend toward reduced *Il-10* mRNA expression in the lung compared to GR^+/+^ mice during PBS and LPS-challenge (Figure 5D). This suggests that increased Opn expression in the lung of GR^dim/dim^ mice might have an impact on *Il-10* mRNA expression. However, in the plasma of GR^+/+^ and GR^dim/dim^ PBS- and LPS-challenged animals only a trend to induced Opn expression was observed, due to high variations in the groups (Supplementary Figure 2A). To assess the effect of impaired GR dimerization in macrophages and their contribution to Opn expression and inflammatory cytokines bone marrow-derived macrophages (BMDM) from GR^+/+^ and GR^dim/dim^ animals were stimulated with LPS. LPS stimulation increased the inflammatory cytokine expression in GR^+/+^ and GR^dim/dim^ BMDMs, however, no genotype difference for the upregulation of *Il-6* expression (Supplementary Figure 2B), *Tnf*α expression (Supplementary Figure 2C), and *iNos* expression (Supplementary Figure 2D) could be detected after LPS treatment. *Opn* expression was not different, however a slight, but not significant, increase could be detected after LPS treatment in GR^+/+^ and GR^dim/dim^ BMDMs with a more pronounced trend in the GR^dim/dim^ BMDMs (Supplementary Figure 2E). This observation is supported by the Opn levels in the supernatant of the LPS-treated GR^dim/dim^ BMDMs, that showed a trend to induced Opn compared to GR^+/+^, however, not significantly different (Supplementary Figure 2F). This suggests that macrophages are not the main source of GR^dim/dim^ dependent regulation of Opn, but they contribute to the Opn induction. Moreover, these data suggest that GR dimerization dependent regulation of Opn *in vivo* might depend on other cells.

In summary, there is possibly a correlation between increased Opn expression in the lung of the GR^dim/dim^ mice that renders them more sensitive during inflammation and therefore, Opn may be a target for the reduced lung compliance.

## Discussion

In the present study, we tested the hypothesis if an impaired glucocorticoid receptor (GR) function, presented as an impaired GR dimerization (GR^dim^), would impair organ function in lipopolysaccharide (LPS)-induced systemic inflammation in mice undergoing intensive care treatment to compensate for LPS-induced cardiovascular depression. We found that GR^dim/dim^ mice challenged with LPS had a significantly increased need of norepinephrine to achieve hemodynamic targets and a more pronounced lactic acidosis during resuscitation measures (“lung-protective” ventilation, fluid resuscitation, and norepinephrine treatment) compared to LPS-challenged GR^+/+^ mice. Most interestingly, GR^dim/dim^ mice challenged with LPS presented with aggravated ALI, shown by a more pronounced impairment of lung mechanics when compared to LPS-challenged GR^+/+^ mice. According to the results of the present study, the impaired lung function in GR^dim/dim^ mice was most likely mediated via an increased endothelial barrier dysfunction, indicated via a reduced expression of sphingosine kinase 1 (*Sphk1*), which was associated with a higher albumin extravasation in lung tissue. Furthermore, the lung injury in GR^dim/dim^ mice was accompanied by an increase in Osteopontin (Opn) levels in lung tissue, which indicated Opn as a marker of lung injury. The impaired lung function in GR^dim/dim^ mice after LPS-challenge was not mediated via the systemic inflammatory response, because we found no differences in cytokine levels between the genotypes, neither in lung tissue (Figure 3) nor in plasma (Table 3).

We previously showed that GR^dim/dim^ mice present a higher mortality in LPS-and cecal ligation and puncture (CLP)-induced systemic inflammation without any resuscitation procedures (18). In the present study, after LPS-challenge GR^dim/dim^ mice had (i) an increased hemodynamic instability indicated by a significantly increased need of norepinephrine, (ii) more pronounced lactic acidosis, and (iii) a reduced lung compliance, which altogether lead to an increased mortality rate despite resuscitative measures. The increased mortality of GR^dim/dim^ mice in the current study (Supplementary Figure 1) confirms that the GR dimer is of importance for survival after challenge with LPS, even when animals receive fluid resuscitation and norepinephrine treatment to achieve target hemodynamics as well as “lung-protective” mechanical ventilation (22, 23).

- ***Hemodynamic instability***: In the present study, GR^dim/dim^ mice presented with an aggravated hemodynamic instability after LPS-challenge, reflected by a significantly increased need of norepinephrine to reach hemodynamic targets (Figure 1). Aggravated hemodynamic instability after LPS-challenge, which also led to increased mortality, has also been reported in mice with an endothelial-specific GR deletion (GR^ECKO^) (30). The increased hemodynamic instability in these mice was accompanied by an increased expression of inducible (iNOS) and endothelial (eNOS) nitric oxide synthase (NOS), which resulted in increased levels of nitric oxide (NO) following LPS-challenge, thus contributing to arterial hypotension. Interestingly, corticosterone levels increased after challenge with LPS, but did not differ between GR^ECKO^ and control mice (30). While an endothelial GR dysfunction induces hypotension, in other studies it is reported that a stimulation of an intact GR via GCs leads to hypertension. In addition, this GC-induced hypertension was reported to be mediated via downregulation of eNOS in rats (31). Therefore, endothelial GR dysfunction leads to NO-induced hypotension, whereas GR stimulation via GCs leads to downregulation of NO synthases, which induces hypertension. Although we did not examine any vascular specific effects of an ubiquitous impairment of GR dimerization, it is likely that the increased hemodynamic instability in GR^dim/dim^ mice has been, at least in part, meditated via a NO-induced vasodilation after LPS-challenge in the present study as reported previously (30). The anesthesia and surgery represent a trauma for the animals and this is most likely the reason for the slight increased mortality of the GR^dim/dim^ mice compared to GR^+/+^ challenged with PBS. We can only speculate about the reasons for this increased instability. Due to the decreased blood pressure in the GR^dim/dim^ mice during the ICU management, it is most likely that either the systemic vascular resistance or the cardiac output is affected. A decreased hemodynamic instability has already been described for mice with an endothelial glucocorticoid receptor knockout (30).
- ***Lactic acidosis***: Additionally, GR^dim/dim^ mice developed lactic acidosis until the end of the LPS-challenge (Table 2). In the present study, GR^+/+^ mice showed an increase in mitochondrial respiration in the liver, which was lacking in GR^dim/dim^ mice (Figure 2). Moreover, GR^dim/dim^ mice presented with a more severe shock and lactic acidosis under highest norepinephrine requirements to counteract arterial hypotension. While shock-induced hypotension causes increased norepinephrine needs to achieve hemodynamic targets, shock-related lactic acidosis originates from disturbed microcirculatory perfusion and/or impaired cellular O_2_ utilization, the later possibly resulting from mitochondrial dysfunction. Moreover, catecholamines *per se* can aggravate both, impaired microcirculatory perfusion and mitochondrial dysfunction, the latter as a result of increased radical formation. Therefore, the lacking increase in mitochondrial respiration in LPS-challenged GR^dim/dim^ mice in the liver and the more pronounced lactic acidosis might be explained by a mitochondrial dysfunction *per se* and/or due to higher norepinephrine doses.
- ***Lung compliance***: The importance of the GR for lung function already becomes apparent during lung maturation *in utero*. Here, GR signaling mediates downsizing of the interstitial mesenchymal tissue compartment. This in turn brings the underlying vasculature into close proximity with the future alveolar airspaces, and enables oxygenation of the blood, therefore allowing survival after birth *ex utero* (32). The importance of the GR for survival after birth becomes apparent even more when studying GR deficient mice. At birth, these GR^−/−^ mice die within a few hours due to respiratory distress, which seems to be mediated through severe lung atelectasis (33). Interestingly, in these GR^−/−^ mice, severe lung atelectasis could not be linked to an impaired surfactant homeostasis. In the present study, we studied mice with an attenuated GR dimerization (GR^dim/dim^), which leads to only partial GR impairment; therefore these mice survive after birth. However, in our model of systemic inflammation via LPS-challenge, GR^dim/dim^ mice presented with impaired lung function, here indicated by a reduced lung compliance (Figure 4A). This reduced lung compliance was accompanied by a clear trend toward an increased albumin extravasation in GR^dim/dim^ mice after LPS-challenge (Figure 4B), indicating an impaired lung barrier function. Independent of the presence or absence of ICU treatment, we (13) and others (34– 36) reported an impaired lung barrier function after LPS-challenge in previous studies. A major mediator of endothelial barrier function in the lung is sphingosine kinase 1 (SphK1). The impaired lung barrier function in our previous study could be linked to a GR dimerization-dependent SphK1 expression in myeloid cells, particularly macrophages, because ablation of the SphK1 gene in the myeloid lineage abolished GC effects on vascular leakage and inflammation (13). In addition, mice with a complete deletion of SphK1 (SphK1^−/−^) are highly susceptible to LPS-induced ALI and exhibit increased lung vascular leakage (37). In the current study, reduced lung compliance after LPS-challenge was accompanied by a significantly lower *Sphk1* expression in GR^dim/dim^ mice (Figure 4C), highlighting the contribution of *Sphk1* expression to mediate a physiological lung function. In addition, there was no change in lung compliance in GR^+/+^ mice despite LPS-challenge but a significant increase of lung *Sphk1* expression compared to PBS-treated GR^+/+^ mice, which also might suggest a beneficial impact of SphK1 in lung function. However, PBS-treated GR^dim/dim^ mice revealed a trend toward a reduced lung *Sphk1* expression, which correlates with the trend, however, not significant, to reduced lung compliance in GR^dim/dim^ animals.

The glucocorticoid receptor is involved in modulating the host response to inflammatory stimuli; therefore, we assessed cytokine mRNA levels in lung tissue (Figure 5) as well as cytokine concentrations in the plasma (Figure 3 and Table 3). Interestingly, we did not find any significant genotype difference in systemic inflammatory mediators in the plasma and lung mRNA expression after LPS-challenge. However, in our previous LPS-induced endotoxic shock model without intensive care management we already described that GR^dim/dim^ mice present increased levels of inflammatory mediators at later time points (18). This could explain the difference to our present findings, where mice were observed for only 6 h after LPS-induced endotoxic shock under intensive care management.

Another inflammatory mediator involved in the pathogenesis of inflammatory diseases is osteopontin (Opn) (38, 39), especially in the lung, as patients with various pulmonary diseases revealed increased lung Opn expression (40–45). Furthermore, in experimental models of lung diseases (asthma, lung injury, lung fibrosis), Opn has a detrimental and functional role (40, 46–50), and, moreover, Opn neutralizing antibody could protect mice against ALI during sepsis (28). We showed that mice with an impaired GR dimerization have elevated Opn protein expression in the lung, the same animals that had a significant decrease in lung compliance after LPS-induced endotoxic shock. The finding that Opn might be a critical regulator of lung compliance is supported by Opn knockout mice showing an increased lung compliance (51). Here we describe, for the first time, that Opn expression in the lung is linked with GR dimerization. Mice lacking GR dimerization had increased Opn expression in the lung, which was accompanied by impaired lung mechanics.

Il-10 is described to be negatively regulated by Opn in LPS-stimulated macrophages, higher Il-10 levels were observed during infections in Opn knockout mice (29). Our data showed a trend to reduced *Il-10* expression in the lung of PBS-treated GR^dim/dim^ mice (Figure 5), and this effect was enhanced in the inflammatory setting. In addition, GR^+/+^ animals revealed a trend to reduced *Il-10* expression in the lung upon inflammation, independent of Opn, suggesting an alternative regulation of *Il-10* that might be mis-regulated in GR^dim/dim^ animals. Possibly, the duration of the GR^dim/dim^ mice in the intensive care management during systemic inflammation was too short to detect more pronounced effects concerning inflammatory cytokines, because our previous results during systemic inflammation (without intensive care management) revealed significant differences at later time points only (18).

In conclusion, impairment of GR dimerization aggravates systemic hypotension and impairs lung function during LPS-induced endotoxic shock in mice. We now demonstrate that the GR dimer is an important mediator of hemodynamic stability and lung function during LPS-induced systemic inflammation. Further studies are warranted to examine if selective activation of the GR dimer may be able to attenuate lung injury during systemic inflammation.

## Data Availability Statement

All datasets generated for this study are included in the article/Supplementary Material.

## Ethics Statement

The animal study was reviewed and approved by the federal authorities for animal research of the Regierungspräsidium Tübingen, Baden-Wuerttemberg, Germany.

## Author Contributions

SV, MW, EC, PR, and JT conceived and designed the study. SV, MW, JP, TM, CH, UW, OM, JV, SK, MG, MF, EC, and UB performed the experiments and organ analysis. AS and PM examined the histology. SV, MW, JP, TM, UW, and OM analyzed the data and interpreted the results. SV, JP, and MW prepared the figures. SV and MW wrote the manuscript. PR and JT revised the manuscript.

### Conflict of Interest

The authors declare that the research was conducted in the absence of any commercial or financial relationships that could be construed as a potential conflict of interest.
