# Supplementary material for: Impaired Glucocorticoid Receptor Dimerization Aggravates LPS-Induced Circulatory and Pulmonary Dysfunction
Source: Front Immunol. 2020 Jan 23;10:3152. doi: 10.3389/fimmu.2019.03152 (PMC6990631; doi:10.3389/fimmu.2019.03152)
Supplement: Supplementary file 1 [file Data_Sheet_1.docx]

Supplementary Material

.


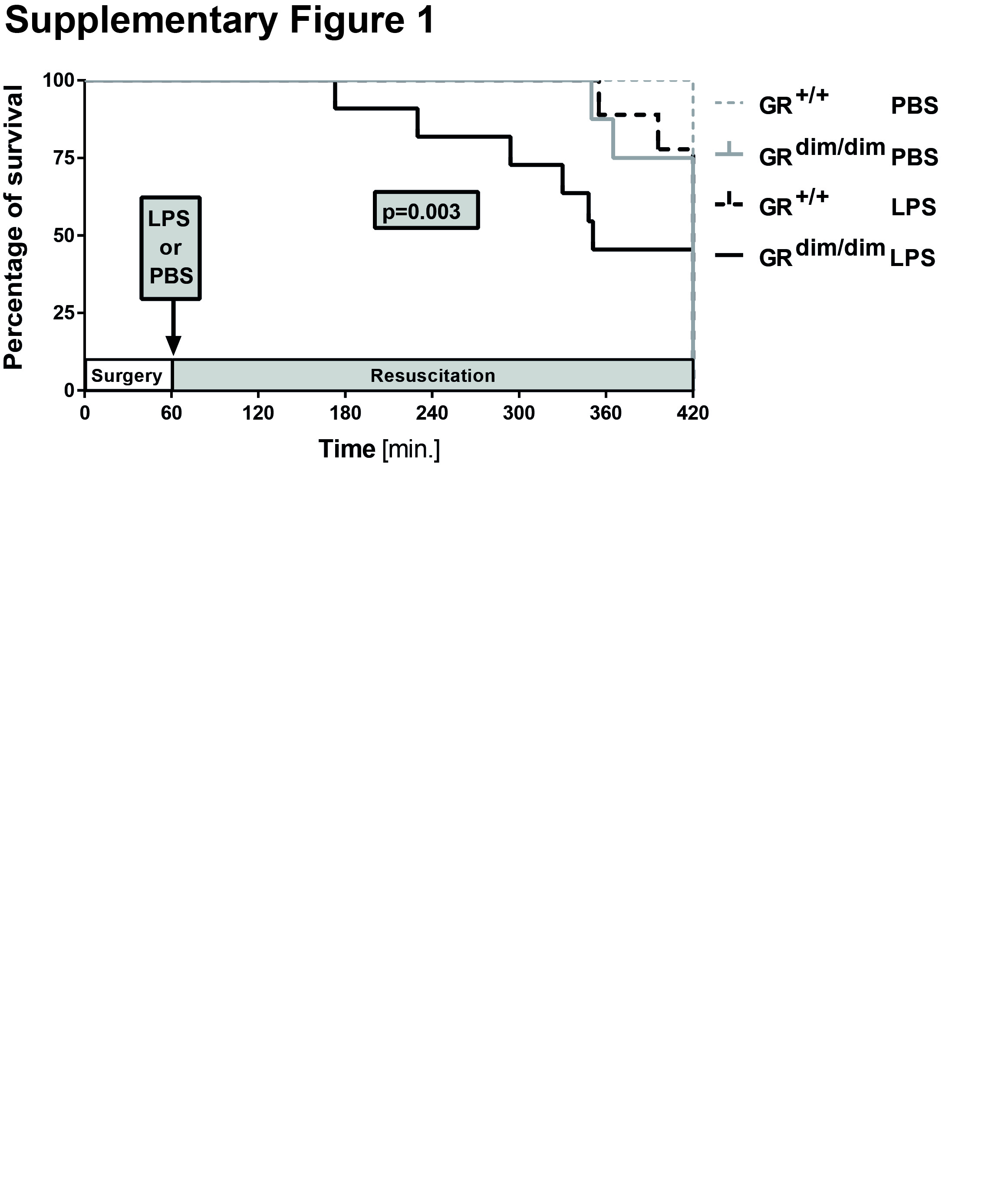


**Supplementary Figure 1.** **Percentage of survival in mechanically ventilated GR^dim/dim^ and GR^+/+^ mice challenged with lipopolysaccharides or vehicle (PBS) and resuscitated (crystalloids, norepinephrine) for six hours.**  LPS=Lipopolysaccharide from Escherichia coli (055:B5), 10mg·kg^-1^ GR^dim^ mice challenged with PBS: n=7, GR^+/+^ mice challenged with PBS: n=8, GR^+/+^ mice challenged with LPS: n=9, GR^dim^ mice challenged with LPS: n=11.


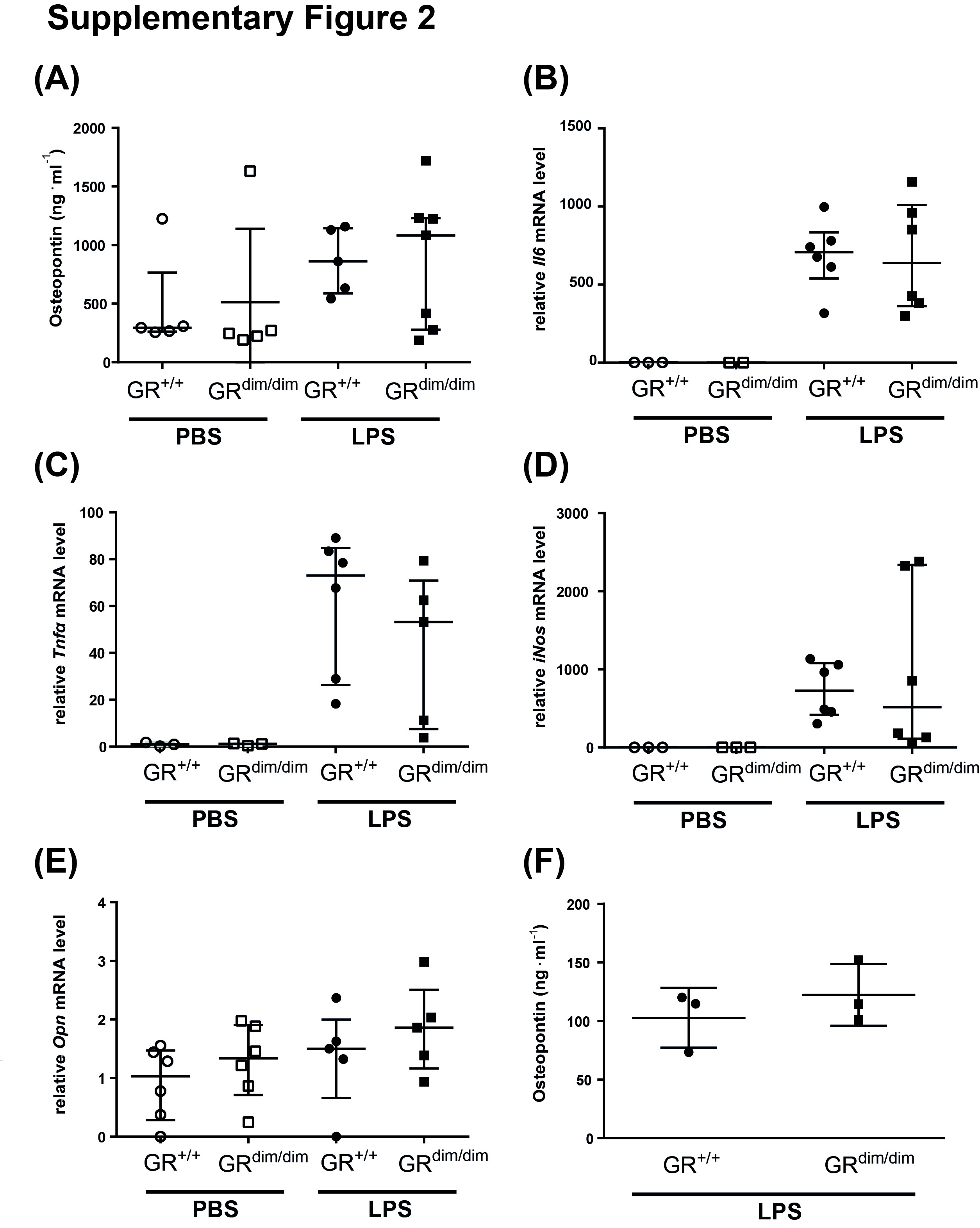


**Supplementary Figure 2.**

**(A)** Plasma Osteopontin in mechanically ventilated GR^dim/dim^ and GR^+/+^ mice intraperitoneally challenged with lipopolysaccharides (LPS) or vehicle (PBS). Osteopontin/secreted phosphoprotein 1 (Opn/Spp1) in plasma of GR^dim/dim^ and GR^+/+^ mice challenged with lipopolysaccharide (LPS) or vehicle (PBS). LPS=lipopolysaccharide from Escherichia coli [055:B5], 10mg·kg^-1^, dissolved in 10µl·g^-1^ phosphate buffered saline (PBS). GR^+/+^ mice challenged with PBS: n=5, GR^dim/dim^ mice challenged with PBS: n=5, GR^+/+^ mice challenged with LPS: n=5, GR^dim/dim^ mice challenged with LPS: n=7. Data is presented as median (25^th^ and 75^th^ percentile and minimum/maximum).

mRNA expression of inflammatory genes in primary bone marrow-derived macrophages (BMDM) isolated from GR^+/+^ and GR^dim/dim^ mice**.** Measurements of relative mRNA level of **(B)** interleukin 6 (*Il-6*), **(C)** tumor necrosis factor α (*Tnfα*), **(D)** inducible nitric oxide synthase (*iNos*) and (**E)** Osteopontin/secreted phosphoprotein 1 (Opn/Spp1) in BMDMs of GR^dim/dim^ and GR^+/+^ mice challenged with PBS or lipopolysaccharide (LPS 100 ng/ml) for 6 hours (B,C,D) or 2 hours (E). (**F)** Osteopontin/secreted phosphoprotein 1 (Opn/Spp1) protein (Elisa) in supernatant of BMDMs from GR^+/+^ and GR^dim^ BMDMs 6 hours after challenged with LPS. GR^+/+^ and GR^dim^ BMDMs challenged with PBS: B, C, D: GR^+/+^ n=3, GR^dim^ n=2-3; E: GR^+/+^ n=6, GR^dim^ n=6. GR^+/+^ and GR^dim^ BMDMs challenged with LPS (100 ng/ml): B, C, D: GR^+/+^ n=6, GR^dim^ n=5-6; E: GR^+/+^ n=5, GR^dim^ n=5; F: GR^+/+^ n=3, GR^dim^ n=3. Data is presented as median (25^th^ and 75^th^ percentile and minimum/maximum).
